# 종합병원 내과 입원 환자에서 카바페넴내성장내세균목 감염증의 발생 현황 및 관련 요인: 후향적 관찰 연구

**DOI:** 10.7586/jkbns.26.012

**Published:** 2026-05-26

**Authors:** Soo-Yeon Park, Hyunjung Kim

**Affiliations:** 1 Graduate School of Nursing Science, Hallym University, Chuncheon, Korea; 1 한림대학교 간호대학원; 2 Department of Nursing, Hallym University Dongtan Sacred Heart Hospital, Hwaseong, Korea; 2 한림대학교 동탄성심병원 간호부; 3 School of Nursing·Research Institute of Nursing Science, Hallym University, Chuncheon, Korea; 3 한림대학교 간호대학·간호학연구소

**Keywords:** Carbapenem-resistant Enterobacterales, Epidemiology, Hospitals, General, Internal medicine, 카바페넴내성장내세균목, 종합병원, 내과, 위험요인

## Abstract

**Purpose:**

This study investigated the incidence, microbiological characteristics, and factors associated with carbapenem-resistant Enterobacterales (CRE) infection among internal medicine patients admitted to general wards at a general hospital.

**Methods:**

This retrospective study was conducted among internal medicine patients admitted to general wards at a general hospital in Korea between January 2023 and December 2024. Among 25,341 admissions, 53 patients with confirmed CRE infection were identified as the case group. A control group comprising 212 patients without CRE infection was selected using simple random sampling at a 4:1 ratio. Multivariable logistic regression analysis was performed to identify factors associated with CRE infection.

**Results:**

The incidence of CRE infection was 2.1 per 1,000 admissions and 0.36 per 1,000 patient-days. Klebsiella pneumoniae was the predominant pathogen, and Klebsiella pneumoniae carbapenemase and New Delhi metallo-β-lactamase were the most common carbapenemase genotypes. Multivariable analysis demonstrated that carbapenem use, acid-suppressive therapy, intensive care unit admission within the previous 3 months, bronchoscopy, and higher Charlson Comorbidity Index scores were significantly associated with CRE infection.

**Conclusion:**

These findings underscore the need for targeted prevention strategies in general wards, particularly for patients with substantial comorbidities and recent healthcare exposures. Implementing robust antimicrobial stewardship programs, strengthening infection control practices for high-risk procedures, and establishing proactive surveillance systems may help reduce the burden of CRE infections in general hospitals.

## 서론

### 1. 연구의 필요성

장내세균목(Enterobacterales)은 다양한 감염을 유발하는 그람음성균으로 전체 의료관련감염의 약 30%를 차지한다. 과거에는 카바페넴 계열 항생제에 대해 높은 감수성을 유지해 왔으나, 카바페넴내성장내세균목(Carbapenem-resistant Enterobacterales, CRE)의 출현으로 인해 항생제 선택이 제한되어 치료가 어려워지고 있다[1]. 카바페넴은 광범위 베타-락탐계 항생제 분해효소(extended-spectrum beta-lactamase, ESBL)를 생성하는 균종의 치료의 핵심 항생제이나, CRE 감염증은 카바페넴뿐만 아니라 대부분의 다른 항생제 계열에도 저항성을 보여 치료 옵션이 polymyxins 및 tigecycline과 같은 제한된 약물에 의존하게 된다. 또한 배양을 통한 확인에 2~3일이 소요되어 적절한 항균 치료가 지연될 수 있으며, 이러한 제한된 치료 옵션과 진단 지연을 포함한 복합적 요인으로 CRE 감염증 환자에서 높은 사망률이 보고되고 있어 임상적으로 중요한 문제로 인식되고 있다[2].

항생제 내성 문제가 적절히 통제되지 못할 경우 2050년까지 전 세계적으로 연간 1,000만 명이 사망할 것이라고 예측되고 있으며[3], 급성기 병원 입원 환자의 7%∼10%에서 발생하고 있는 의료관련감염 중에서 약 4분의 1은 항생제 내성균에 의해 발생하는 것으로 보고된다[4]. 특히 CRE 감염증은 코로나19 팬데믹 기간 동안 뚜렷한 증가 양상을 보여 2020년에는 전년 대비 35.0% 증가하였고, 2022년에도 팬데믹 이전 수준을 지속적으로 상회하고 있다. 이는 팬데믹 기간 동안 입원 기간 증가와 항생제 사용 증가, 감염관리 자원의 제한 등 의료환경의 변화와 함께 항생제 내성 감염의 증가로 인한 것으로 보고되고 있다[5].

국내 질병관리청의 2022년 감염병 감시 연보에 따르면 CRE 감염증 발생 중 80세 이상이 36.2%로 가장 많았으며, 전체 발생 건수의 약 82.6%가 60세 이상 고령층에서 발생하였다[6]. CRE 균혈증 환자를 대상으로 한 선행연구에서는 20.5%의 대상자가 중증 또는 만성 신장질환을, 27.4%는 면역억제 상태를 동반하고 있었으며, 24.6%와 31.8%의 대상자가 최근에 침습적 시술과 항생제 노출을 경험하였다[7]. 내과 환자군은 고령, 면역저하 상태, 만성질환, 침습적 시술 경험 등 이러한 위험 요인을 동시에 보유한 CRE 감염의 주요 발생 집단이다. 미국의 다기관 연구[2]에서는 CRE 감염 환자의 평균 연령이 65세였으며, 약 50%가 암 치료 또는 고형 장기 이식 등으로 인한 면역저하 상태였다. 주요 동반질환으로는 당뇨병(30%)과 심부전(17%)이 높은 비중을 차지하였고, 상당수 환자가 내시경적 역행 담췌관조영술(endoscopic retrograde cholangiopancreatography, ERCP)과 같은 침습적 시술을 경험한 것으로 보고되어, 내과 환자가 CRE 감염에 취약한 임상적 특성을 가지고 있음을 시사한다.

국내 종합병원은 전체 병상의 17.6%를 차지하며[8,9], 2023년 전국 CRE 감염증 사례 중 43.3%가 종합병원에서 보고되었다[10]. 경기도 보건환경연구원 분석에서도 종합병원이 60.9%로 의료기관 유형 중 CRE 발생률이 가장 높은 것으로 확인되었다[11]. 상급종합병원의 CRE 신고율은 감소하는 반면, 종합병원은 약 45% 수준을 지속적으로 유지하고 있으며[12], 종합병원은 의료전달체계에서 1차와 3차 의료기관을 연결하는 중간 단계에 위치하여 환자 회송과 전원이 빈번하므로 CRE 감염관리 전략의 핵심 기점으로 중요한 위치를 차지한다[13]. 그러나 국내 다제내성균(multidrug-resistant organisms, MDRO) 관리체계는 상급종합병원과 중환자실 중심으로 구축되어 있어, 종합병원 일반병동 내과 입원 환자를 대상으로 한 근거 기반 관리 전략은 상대적으로 부족한 실정이다[14].

그러므로 종합병원 일반병동에 입원한 내과 환자를 대상으로 CRE 감염증의 발생 양상과 관련요인을 규명하는 것은, 종합병원 수준에서 실제 적용 가능한 감염관리 전략을 수립하고 지역사회 및 의료기관 간 CRE 확산을 차단하기 위한 과제라 할 수 있다. 이에 본 연구는 종합병원 일반병동 내과 입원 환자를 대상으로 CRE 감염증의 발생 현황과 역학적 특성을 파악하고 CRE 감염증 발생과 관련된 요인을 규명하고자 한다. 이를 통해 향후 의료기관의 감염 예방 및 관리 전략 수립을 위한 과학적 근거를 제공하고자 한다.

### 2. 연구의 목적

본 연구는 종합병원에 입원한 내과 환자를 대상으로 CRE 감염증의 분리 균 특성과 CRE 감염증 발생과 관련된 요인을 규명하는데 목적이 있다. 구체적인 목적은 다음과 같다.

1) 종합병원에 입원한 내과 환자의 CRE 감염증 발생률과 분리균의 특성을 파악한다.

2) 종합병원에 입원한 내과 환자의 특성에 따른 CRE 감염증 발생률의 차이를 파악한다.

3) 종합병원에 입원한 내과 환자의 CRE 감염증 발생과 관련된 요인을 분석한다.

## 연구 방법

### 1. 연구 설계

본 연구는 종합병원 내과병동에 입원한 환자를 대상으로 CRE 감염증 발생률을 파악하고, CRE 감염증 발생의 관련 요인을 알아보기 위한 후향적 조사연구로 STROBE (Strengthening the Reporting of Observational studies in Epidemiology) reporting guideline을 준수하였다.

### 2. 연구 대상

본 연구의 대상자는 2023년 01월부터 2024년 12월까지 한림대학교 동탄성심병원의 일반 병동에 내과 진료과로 입원한 성인 환자를 대상으로 하였다. 해당 병원은 약 650병상을 보유하고 있으며, 7개의 중환자실과 12개의 일반병동을 운영하고 있다. 내과는 소화기내과, 호흡기내과, 감염내과, 순환기내과, 혈액종양내과, 신장내과, 내분비내과 등 총 7개의 세부 전문분야로 구성되어 있다.

연구기간 동안 12개 일반병동에 내과 입원 환자로 등록된 만 18세 이상 성인 25,341명 중 CRE 감염이 확인된 53명을 감염군으로 분류하였다. 사례 수가 제한적인 점을 고려하여 통계적 검정력을 확보하기 위해 선행 연구[15,16]를 참조하여 감염군 대비 4배수의 비감염군을 선정하였다. 비감염군은 동일 연구기간 동안 동일 모집단에 속하는 내과 입원 환자 중 CRE 감염이 확인되지 않은 환자를 대상으로 하였으며, 감염군 사례 수의 4배수에 해당하는 환자 212명을 단순 무작위추출(simple random sampling) 방식으로 선정하였다. 이와 같은 절차를 통해 최종적으로 총 265명의 연구 대상자가 연구에 포함되었다. 본 연구에서 CRE 감염증은 질병관리청의 정의에 따라 카바페넴 계열 항생제에 최소 한 가지 이상 내성인 장내세균목의 균종에 의한 감염질환을 CRE 감염증으로 정의하였으며[17], 이에 근거한 감염군과 비감염군의 세부 선정기준은 다음과 같다. 감염군의 선정기준은 만 18세 이상 성인으로 일반병동에 입원한 내과 환자 중 입원 48시간 이후 검사한 임상검체(전혈, 대변, 직장도말, 소변, 가래, 뇌척수액, 농양, 기관지 흡입물, 기타)에서 카바페넴계 항생제(ertapenem, meropenem, doripenem, imipenem)에 최소 한 가지 이상 내성인 장내세균목의 균종이 배양된 환자이다[17-19]. 비감염군의 선정기준은 동일 기간 내 일반병동에 입원한 내과 성인 환자 중 입원하여 입원 48시간 이후 검사한 배양검사결과가 음성인 환자이다. 제외기준으로는 첫째, 입원 48시간 이내에 시행한 배양검사에서 CRE가 분리된 경우는 입원 전 이미 감염이 존재하였거나 잠복 중이었던 것으로 간주하므로 제외하였다[18]. 둘째, 동일 환자에서 연구 기간 중 CRE 발생 후 재입원한 경우는 최초 입원 시의 첫 발생만을 포함하였다. 셋째, 동일 입원 기간 내 CRE가 2회 이상 검출된 경우에는 첫 번째 검출만을 분석에 포함하였다.

### 3. 연구 도구

질병관리청의 의료관련감염병 관리지침과 선행연구[17,18,20-24]를 기반으로 CRE 감염증이 발생한 환자의 특성에 대한 자료 수집을 위해 본 연구자가 개발한 ‘CRE 감염증 발생 역학적 특성 조사지’를 사용하였다. 본 연구의 조사지는 인구학적 특성, 질병 관련 특성, 침습적 치료 관련 특성, 치료 및 항생제 사용 관련 특성, 다제내성균 관련 특성으로 구성되며 7개 내과의 다빈도 치료 내용을 포괄하고 있다.

#### 1) 인구학적 특성

인구학적 특성은 성별, 나이, 재원기간, 퇴원 시 생존 여부, 입원경로로 구성하였다. 입원경로는 의료법에 근거한 의료기관과 노인장기요양법에 근거한 장기요양기관을 포함하였고, 외래를 경유하여 입원한 경우는 지역사회에서 유입된 것으로 간주하여 지역사회로 분류하였다.

#### 2) 질병관련 특성

질병 관련 특성은 주진단명, Charlson Comorbidity Index (CCI)에 기반한 동반질환과 점수, 입원 시 욕창 보유 여부와 영양상태, 3개월 이내의 수술력과 중환자실 입원여부를 포함하였다.

#### 3) 치료적 특성

치료적 특성은 약물과 침습적 기구 사용 및 침습적 시술의 범주로 7개 내과에서 시행되는 주요 내용을 포함하였다. 약물과 침습적 시술은 최근 3개월 이내 치료 이력이 있는 것으로 한정하였다. 약물에는 항생제로 페니실린, 3,4세대 세팔로스포린, 카바페넴, 플루오로퀴놀론, 글리코펩타이드 계열을 포함하며, 기타 분류로 아미노글리코사이드, 테트라사이클린, 마크로라이드 계열을 포함하였다. 총 비경구 영양, 스테로이드, 위산 억제제, 화학요법, 네뷸라이저 치료도 포함하였다. 침습적 시술은 기관지내시경, 위장관 내시경, ERCP, 심혈관 조영술, 혈액투석과 경피적생검을, 침습적 기구는 기관 절개관, 인공호흡기, 비위관, 혈관 카테터, 요로 배액관과 배액 카테터를 포함하였다.

#### 4) 다제내성균 관련 특성

다제내성균 관련 특성에서는 세부 균주, carbapenemase-producing Enterobacterales (CPE) 분리여부를 포함하였다. CPE 분리시에는, CPE 유전자형인 Klebsiella pneumoniae carbapenemase (KPC), New Delhi metallo-β-lactamase (NDM), OXA-type carbapenemase (OXA), Other로 세부로 분류하였다. 기타 다제내성균은 vancomycin-resistant Enterococcus (VRE), extended-spectrum β-lactamase (ESBL), multidrug-resistant Pseudomonas aeruginosa (MRPA), multidrug-resistant Acinetobacter baumannii (MRAB)로 나누고 CRE 감염증 3개월 이전에 분리된 경우로 한정하였다.

### 4. 자료 수집

본 연구의 자료는 한림대학교 동탄성심병원에서 사용 중인 Smart Clinical Data Warehouse를 활용하여 후향적으로 수집하였다. 2023년 01월 01일부터 2024년 12월 31일까지 7개 내과 총 25,341명 입원환자 중 53,271건의 미생물 배양검사 결과를 수집하고 입원 48시간 이후 검사한 배양검사결과가 카바페넴계 항생제에 최소 한 가지 이상 내성인 장내세균목의 균종이 배양된 환자와, 동일한 시점의 배양검사에서 음성으로 확인된 환자만을 분석 대상으로 포함하였다. 이후 전자 의무기록을 후향적으로 검토하여 연구자가 개발한 CRE 감염증 역학적 특성 조사지를 사용하여 자료수집을 시행하였다. 일반적 특성은 간호정보조사지와 의사의 퇴원 요약지에서 추출하였으며 질병 관련 특성은 진단 코딩정보지로 주진단명을 추출하고 입원기록지로 CCI에 기반한 동반질환과 점수를 연구자가 직접 계산하였다[24]. 입원 시 욕창 보유는 욕창사정관리 기록지에서 영양상태는 영양검색기록지, 3개월 이내의 수술력과 중환자실 입원여부는 간호정보조사지를 통해 추출하였다. 침습적 기구 적용은 간호활동기록지, 중환자실 간호기록지를 통해, 치료적 특성 및 약물 사용은 의사지시서를 통해 추출하였다. MDRO균 관련 특성은 일반미생물 검사결과지를 통해 추출하였다.

### 5. 자료 분석

자료 분석은 R version 4.3.0 (R Foundation for Statistical Computing, Vienna, Austria)을 이용하였으며, 양측검정에서 *p* < .050을 통계적으로 유의한 것으로 해석하였다.

1) 종합병원에 입원한 내과 환자의 CRE 감염률은 입원환자(admissions) 1,000명 및 1,000 재원일수(patient-days)를 기준으로 산출하였다. 입원환자 기준은 (CRE 감염 건수/ 총 입원환자 수) × 1,000으로, 재원일수 기준은 (CRE 감염 건수/ 총 재원일수) × 1,000으로 산출하였다.

2) 연구대상의 일반적 특성, 치료적 특성, CRE 분리 균의 특성은 기술통계를 사용하였으며, 연속형 변수는 Shapiro-Wilk test로 정규성을 검정한 후 정규분포를 따르지 않는 경우 중앙값과 사분위수 범위(interquartile range)로 표시하였다.

3) CRE 감염증 유무에 따른 연구대상의 특성의 차이 분석은 범주형 변수는 chi-square test를 사용하였으며, 기대빈도가 5 미만인 셀이 있는 경우 Fisher’s exact test를 적용하였다. 연속형 변수는 정규분포를 따르지 않는 경우 Mann-Whitney U test를 사용하여 비교하였다.

4) CRE 감염증 발생과 관련된 요인을 확인하기 위해 단변량 분석 후 유의한 변수들을 포함한 다변량 로지스틱 회귀분석을 시행하였다. 회귀분석은 2단계 접근법을 사용하였으며, 단변량 분석에서 *p* < .050을 나타낸 변수를 다변량 모델에 포함시켰다. 포함된 변수는 성별, 입원 경로, CCI 점수, 영양상태, 최근 3개월 이내 중환자실 입원력, 항생제 사용 여부, 위산분비억제제(proton pump inhibitor, PPI), 총 비경구 영양, 네뷸라이져 사용, 그리고 기구 관련 침습적 처치 변수들이었다. 연속형 변수에 대해서는 두 군 간 비교 분석을 위해 정규성 검정을 시행하였으며, 정규성을 만족하지 않는 경우 비모수 검정을 적용하였다. 다변량 로지스틱 회귀분석에서는 연속형 변수를 원자료 형태로 투입하였고, 범주형 변수는 이분형(dummy) 변수로 변환하여 분석하였다. 모델의 적합도는 Hosmer-Lemeshow goodness-of-fit test로 평가하였으며, 모델의 설명력은 Cox & Snell R^2^와 Nagelkerke R^2^를 사용하였다. OR (odds ratio)이나 adjusted OR이 5.0 이상인 경우 강한 관련 요인, OR이 2.0~4.9인 경우 중등도 관련 요인으로 해석하였다[25–27].

### 6. 윤리적 고려

본 연구는 한림대학교 동탄성심병원 임상연구심의위원회 심의를 받아 승인 후 시행하였다(HDT IRB 2025-06-012-001). 대상자의 전자의무기록을 후향적으로 조사하는 연구로 대상자의 동의는 면제되었다. 연구대상자는 무기명으로 부호화하여 코딩하였으며 개인정보와 의무기록은 프로그램 암호화를 통해 비밀을 보장하여 개인을 식별할 수 없도록 하였다. 모든 자료는 비밀번호가 설정된 개인 컴퓨터에 보관하여 개인정보를 보호하였다.

## 연구 결과

### 1. 종합병원 일반병동 내과환자의 CRE 감염증 발생률

본 연구의 선정기준에 해당하는 대상자 25,341명 중 53명이 CRE 감염으로 확인되었으며, 이는 1,000 입원환자당 2.1명의 발생률에 해당하였고, 총 재원일수 145,772일을 기준으로 하면 1,000 재원일수 당 0.36건의 발생률이 확인되었다(Table 1).

### 2. 종합병원 일반병동 내과 환자의 CRE 감염증 환자의 분리 균 특성

본 연구에서 CRE 감염증 환자 53명을 분석한 결과, 분리된 균종 중 *Klebsiella pneumoniae*가 44건(82.9%)으로 가장 많았으며, 카바페넴분해효소 생성 장내세균목(CPE)은 30명(56.6%)에서 확인되었다. CPE 양성 환자를 대상으로 카바페넴분해효소 유전자형을 분석한 결과, KPC 유전자가 22명(73.3%)으로 가장 흔하였고, NDM 유전자는 6명(20.0%)에서 확인되었다. MDRO는 전체 환자 중 8명(15.1%)에서 동반되어 확인되었으며, 그 중 ESBL이 4명(50.0%)으로 가장 많았다(Table 2).

### 3. 대상자의 특성에 따른 CRE 감염증 발생의 차이

대상자의 일반적 특성 중 남성이 37명(69.8%)으로 여성에 비해 감염군에서 유의하게 많은 반면(*p* = .033), 나이에서는 차이를 보이지 않았다. 질병 관련 특성 중 욕창과 3개월 이내 수술 이력을 제외한 진단명(*p* < .001), 입원 경로(*p* = .010), CCI 점수(*p* < .001), 영양상태(*p* < .001), 3개월 이내 중환자실 입원력(*p* < .001)에 따라 CRE 감염증 발생에 유의한 차이를 보였다(Table 3).

약물 치료 중에서는 Penicillin (*p* < .001), Carbapenem (*p* < .001), Fluoroquinolone (*p* = .013), Glycopeptide (*p* < .001), Tetracycline (*p* = .039) 항생제와 총 비경구 영양(*p* < .001), 위산 억제제(*p* < .001), 네뷸라이져(*p* < .001) 사용 여부에 따라 CRE 감염증 발생에 유의한 차이가 있었다. 침습적 기구 중에서는 기관 절개관(*p* = .026), 인공호흡기(*p* < .001), 비위관(*p* < .001), 혈관 카테터(*p* < .001), 요로 배액관(*p* < .001)과 배액 카테터(*p* = .011)를 가지고 있는 경우와 기관지내시경(*p* < .001)을 시행한 경우 유의한 차이를 보였다(Table 4).

### 4. CRE 감염증 발생에 관련된 요인

로지스틱 회귀분석의 단변량 분석에서는 여성(OR 0.48, 95% confidence interval [CI]: 0.25–0.91), 영양 상태의 중간위험군(OR 2.62, 95% CI: 1.10–6.22)과 고위험군(OR 7.89, 95% CI: 2.66–23.37), 최근 3개월 이내 중환자실 입원력이 있는 경우(OR 11.91, 95% CI: 5.38–26.38)와, fluoroquinolone (OR 2.69, 95% CI: 1.28–5.63), glycopeptide (OR 11.07, 95% CI: 4.86–25.21), penicillin (OR 2.80, 95% CI: 1.51–5.18), carbapenem (OR 12.52, 95% CI: 6.14–25.52), PPI (OR 7.21, 95% CI: 3.50–14.83), 총비경구영양(OR 5.83, 95% CI: 2.90–11.71), 네뷸라이저 치료(OR 6.75, 95% CI: 3.12–14.64), 기관지내시경(OR 9.40, 95% CI: 4.09–21.59)을 받은 경우, 요로 배액관(OR 3.38, 95% CI: 1.80–6.34)과 혈관 카테터(OR 4.71, 95% CI: 2.50–8.87)를 가지고 있는 경우에서 CRE 감염증 발생위험이 유의하게 높았다. 또한 CCI 점수 (OR 1.30, 95% CI: 1.15–1.47)가 증가할수록 감염 위험이 증가하였다.

다변량 로지스틱 회귀분석 결과, CRE 감염증 발생의 강한 관련 요인으로는 carbapenem계 항생제(adjusted OR 8.10, 95% CI: 2.02–32.41)와 PPI 사용(adjusted OR 7.62, 95% CI: 2.27–25.56), 최근 3개월 이내 중환자실 입원력이 있는 경우(adjusted OR 6.23, 95% CI: 1.27–30.58)와 기관지내시경 시행(adjusted OR 5.31, 95% CI: 1.07–26.42)으로 나타났다. 중등도 관련 요인으로는 페니실린계 항생제 사용이 확인되었으며(adjusted OR 3.64, 95% CI: 1.21–10.92), CCI 점수는 점수가 1점 증가할 때마다 CRE 감염 위험이 약 1.36배 증가하는 것으로 확인되었다(adjusted OR 1.36, 95% CI: 1.10–1.69).

모형의 적합도 평가에서 Hosmer–Lemeshow 검정 결과 모형의 적합도는 양호하였으며(χ^2^ = 3.21, *p* = .920), Cox & Snell R^2^는 .43, Nagelkerke R^2^는 .67로 본 다변량 로지스틱 회귀모형의 설명력은 비교적 높은 수준인 것으로 나타났다(Table 5).

## 논의

본 연구는 국내 종합병원 일반병동에 입원한 내과 환자의 CRE 감염증 발생 현황과 관련 요인을 분석하였다. 2023년 1월부터 2024년 12월까지 입원한 내과 환자 25,341명 중 53명(0.21%)이 CRE 감염증으로 확인되었으며, 이는 1,000 입원환자당 2.1명, 1,000 patient-days당 0.36건의 발생률에 해당하였다. 이 수치는 2024년 국내 상급종합병원 노숙자 병동을 대상으로 한 연구에서 보고된 1,000 patient-days당 1.14건의 CRE 발생률[28]보다 낮고, 2021년 국내 상급종합병원 중환자실을 대상으로 한 연구에서 보고된 4.3건의 CRE 발생률[29]에 비해서도 낮았다. 또한 2023년 국내 종합병원에서 입원 병동을 구분하지 않고 시행된 연구에서 보고된 1.9건의 CRE 발생률[30]보다도 낮았다. 국외 연구와 비교하였을 때, 2025년 인도 다기관 연구에서는 0.86건의 CRE 감염증 발생률이 보고되었으며[31], 2021년 미국 장기요양시설을 대상으로 한 연구에서는 0.46건의 CRE 발생률이 확인되었다[32]. 본 연구는 종합병원 일반병동을 대상으로 한 연구로 선행 연구들과 정확히 비교하기는 어려우나 종합하면, 본 연구의 내과병동 CRE 발생률(0.36 건/1,000 patient-days)은 국내외 비중환자 환경에서 보고된 범위 (0.2∼1.1 건/1,000 patient-days) 내에 해당하며[30-32], 중환자실 또는 특수병동과 같은 고위험 환경(1.5∼6.0건/1,000 patient-days)[28,29]에 비해 상대적으로 낮은 발생률을 나타냈다. 국내에서는 CRE 감염증 발생의 증가로 인해 입원 시 고위험군 선별검사, 입원 중 CRE 발생 시 즉각적인 격리 및 접촉주의 적용, 동일 병실 환자에 대한 반복 검사, 그리고 집단 발생 시 역학조사와 능동감시배양 확대를 시행하는 질병관리청의 의료관련감염병 관리지침[20]의 수행이 강조되고 있으며, 의료기관 평가의 지표로도 활용되고 있다. 본 연구에서 일반병동의 CRE 발생률이 상대적으로 낮게 관찰된 것은 일반병동 환자의 낮은 중증도와 침습적 처치 빈도라는 환자군 특성에 더해, 이러한 표준화된 대응 체계가 일반병동 환경에서도 CRE의 전파를 효과적으로 차단하는 데 기여함으로써 적극적으로 관리지침이 시행되기 전에 시행된 선행 연구들에 비해 본 연구에서 CRE 발생률이 낮게 관찰되었을 가능성이 있다.

본 연구에서 주요 분리균은 *Klebsiella pneumoniae* (82.9%)가 대부분을 차지하였다. 이는 2023년 질병관리청의 전국전수조사 결과에서 보고된 *K. pneumoniae* (68.1%)와 *E. coli* (17.4%)가 전체 CRE의 85.5%를 차지하였다는 결과와 유사하므로[21], 국내 CRE 감염증의 주요 원인균이 일관되게 *K. pneumoniae*임을 확인하였다. 병동 유형별로 살펴보면, 2021년 국내 상급종합병원 중환자실을 대상으로도 주요 분리균은 *K. pneumoniae*였으며[29], 2024년 국내 상급종합병원 노숙자 병동을 대상으로 한 연구에서도 *K. pneumoniae*가 주요 분리균으로 확인되어 병동 유형이나 환자 특성과 관계없이 동일한 균종이 우세함을 보여주었다[28]. Carbapenemase 생성 여부를 분석한 결과, 본 연구에서는 전체 CRE 감염증의 56.6%에서 CPE 생성이 확인되었고, 주요 유전자형은 KPC (73.3%)와 NDM (20.0%)이었다. 2021년 질병관리청의 보고에서는 전국 분리 CRE 중 83.0%가 CPE이었고, 그 중 KPC-2형이 79.4%, NDM형이 15.2%로 확인되었다[33]. 또한 2022년 경기도 내 의료기관에서 분리된 1,243건의 CRE를 분석한 연구에서도 66.8%가 CPE로 확인되었으며, 이 중 KPC 유전자가 44.3%, NDM 유전자가 20.4%를 차지하는 것으로 보고하였다[34]. 비율의 차이는 있으나, 세 연구 모두 KPC형이 국내 CRE의 주요 내성기전으로 분포하는 공통 경향을 보여주었다. CPE는 플라즈미드를 통한 유전자 전달이 가능하여 전파력이 높고, 병원 환경 내에서 빠른 확산과 집단 발생을 유발할 수 있어 일반병동 환경에서도 적절한 감시와 전파 차단 조치가 동반되지 않을 경우, 병원 내 전파가 지속되거나 확대될 위험이 있다[22]. 따라서 일반병동을 포함한 병원 환경에서는 CPE에 대한 조기 감시와 신속한 발견을 기반으로 한 격리 및 접촉주의의 즉각적인 적용이 중요하며, 고위험 환자를 대상으로 한 선별검사와 감시배양을 포함하는 포괄적인 감염관리 전략이 시행될 필요가 있다. 이러한 접근은 일반병동에서의 CRE 전파를 예방하고, 병원 내 감염 확산을 효과적으로 차단하는 데 기여할 것으로 판단된다. 요약하면, 본 연구 결과는 병원 유형별 연구[11]에서 제시된 CRE 균종 분포 경향과 일치하고 *K. pneumoniae*가 주요 우세 균종으로 나타났으며, 2024년 질병관리청의 전국 전수조사 결과에서 확인된 KPC형 중심의 내성 유전자형 경향과 부합한다[21]. 이는 종합병원 일반병동 내과 환자에서도 국내 CRE의 양상이 반영되고 있음을 시사하며, 향후 기관 규모를 불문한 지속적 감시와 병동 단위의 감염관리 강화가 필요함을 강조한다.

본 연구에서 종합병원 내과 입원 환자의 CRE 감염증 발생에 관련된 요인으로는 카바페넴계 항생제 사용, PPI 사용, 최근 3개월 이내 중환자실 입원력, 그리고 기관지내시경 시행이 다변량 로지스틱 회귀분석에서 각각 8.10, 7.62, 6.23, 5.31의 OR 5 이상에 해당하는 강한 관련 요인으로 확인되었으며, 이러한 결과는 국내외 여러 선행연구들과 일관된 양상을 보인다. 국외 연구에서는 카바페넴계 항생제 사용 시 CRE 감염 위험이 약 12.5배 증가한다고 보고하였으며[34], 국내 대규모 코호트 연구에서도 카바페넴계 항생제 사용이 CRE 발생의 주요 관련 요인으로 확인되었고, 특히 최근 3개월 이내 meropenem 사용 이력은 CRE 발생의 강력한 예측인자로 제시되었다[36]. 또한 국외 다기관 연구[2]와, 국내 상급종합병원에서 수행된 연구들[2,29]에서도 카바페넴계 항생제 또는 광범위 항생제 사용이 CRE 감염의 주요 결정인자로 보고되었다. 이는 카바페넴계 항생제의 과도한 사용이 장내 정상균총을 파괴하고 내성균의 선택적 증식을 유발하며, 내성 유전자가 다른 내성 유전자들과 함께 플라즈미드를 통해 전달됨으로써 다제내성균의 출현과 확산을 촉진한다는 기전으로 설명될 수 있다[35,36]. 이에 따라 항생제 관리 프로그램(antimicrobial stewardship program, ASP)의 강화가 필수적이며, 특히 카바페넴계 항생제의 적정 사용은 병원 내 CRE 감염증 발생을 감소시킬 수 있는 핵심 전략으로 제시된다.

이에 더하여, PPI 사용 역시 CRE 감염의 강한 관련 요인으로 확인되었다. PPI는 위내 산도를 저하시켜 병원성 세균의 생존 가능성을 증가시키고, 장내 미생물 다양성을 감소시키는 것으로 알려져 있다[36]. 특히 PPI와 카바페넴계 항생제를 병용할 경우 CRE의 장내 정착 및 증식 위험이 더욱 증가할 수 있음이 보고되었으며[37], 이는 PPI 단독 사용보다 항생제와 병용 시 CRE 감염증 발생 위험이 유의하게 높아진다는 점을 시사한다. 카바페넴계 항생제 노출이 CRE 감염증 발생의 주요 관련 요인으로 보고되어 온 점을 고려하면, 이러한 병용 상황에서는 CRE 감염 위험이 더욱 증폭될 가능성이 있다[37]. 따라서 향후 감염관리 전략에서는 ASP를 항생제 사용 관리에 국한하지 않고, PPI의 적정 사용 평가와 불필요한 투여 제한을 포함하는 통합적 stewardship 체계로 확장할 필요가 있다[38].

최근 3개월 이내 중환자실 입원력 또한 CRE 감염 위험을 유의하게 증가시키는 요인으로 확인되었다. 이러한 결과는 2022년 국외 3차 대학병원 연구에서 CRE 보균자가 감염으로 전환되는 데 중환자실 입원력이 중요한 역할을 한다고 보고한 결과와 유사하다[39]. 또한 2022년 국외 다기관 연구에서도 중환자실 입원력이 CRE 보균 위험을 약 3배 증가시키는 것으로 나타나[40], 중환자실이라는 고위험 의료 환경이 CRE 감염 발생에 중요한 영향을 미친다는 점을 본 연구 결과와 함께 뒷받침한다.

본 연구에서는 기관지내시경 시행 또한 CRE 감염의 강한 독립 관련 요인으로 확인되었다. 이는 기관지내시경의 구조적 특성상 재처리 과정에서 미세한 오염이 잔존할 가능성이 있으며, 이로 인해 CRE를 포함한 다제내성균의 전파 위험이 증가할 수 있음을 시사한다[41]. 본 연구에서는 기관지내시경 기구의 실제 오염도나 재처리 과정의 적정성을 직접 평가하지 못하였다는 한계가 있으나, 다수의 선행연구에서 기관지내시경 재처리 미흡이 MDRO 전파의 주요 관련 요인으로 지목되어 왔다. 따라서 특히 기관지내시경과 네뷸라이저 등 호흡기 관련 시술 시에는 재처리 과정의 철저한 준수와 감염관리 지침의 강화가 필요하다.

마지막으로, CCI 점수가 CRE 감염증 발생의 유의한 관련 요인으로 확인되었다. 이는 기저질환 부담이 클수록 CRE 감염 위험이 높아진다는 것을 의미하며, 이러한 경향은 선행연구에서도 일관되게 보고되었다. 국내 연구들에서 중환자실 환자군에서 CCI 점수가 높을수록 CRE 감염 위험이 증가한다고 보고하였으며[29,42], 국외 연구에서는 높은 CCI 점수가 CRE 감염 및 불량한 임상 결과와 유의한 관련이 있음을 보고하여[27], 본 연구의 결과를 뒷받침한다. 이러한 근거들을 종합할 때, CCI 점수는 단순한 동반질환 지표를 넘어 CRE 감염 고위험군을 선별하는 중요한 예측 지표로 활용될 수 있다[43]. 특히 CCI ≥ 4와 같은 고위험 기준을 적용할 경우, 감염관리 자원을 모든 환자에게 일률적으로 분산하기보다 고위험군을 중심으로 한 표적 감염예방 전략을 집중적으로 적용할 수 있을 것으로 여겨진다[44].

영양 상태는 비록 본 연구의 다변량 회귀분석에서는 통계적으로 유의하지 않았으나 단변량 분석에서는 유의한 관련요인으로서, 영양상태가 나쁠수록 CRE 감염증 발생 위험이 높은 것으로 나타났다. 영양불량은 IgA의 생산 감소, 및 장점막 투과성 증가로 인한 장내 방어기능 저하와 CD4 T 세포의 기능 조절 이상을 통해 면역체계 손상을 초래함으로써 감염 위험을 증가시키는 것으로 보고되고 있다[45]. 그러므로 입원 시 표준화된 영양 선별 도구를 활용한 체계적 영양상태 사정과 적극적인 영양 중재가 감염관리와 함께 통합적으로 수행될 필요가 있다.

위와 같이 본 연구에서 확인된 CRE 감염증의 관련 요인은 중환자실이나 상급종합병원 중심의 선행연구에서 보고된 동일한 요인들이 일반내과 병동 환경에서도 유효하게 작용함을 확인하였다는 점에서 의미가 있다. 특히 중환자실 입원력이 일반병동 환자에서도 CRE 발생의 강한 관련요인으로 확인된 것은, 중환자실과 일반병동 간 환자 이동 시 감염관리의 연속성이 중요함을 보여준다. 또한 기관지내시경이 관련 요인으로 확인된 점은 중환자실 환경에서 주로 논의되던 침습적 처치 관련 위험이 일반병동에서도 실재함을 의미하며, 병동 유형에 관계없이 침습적 기구의 철저한 재처리와 감염관리 지침 준수가 필요함을 강조한다.

본 연구는 상대적으로 연구가 부족하였던 종합병원 일반내과 병동에서 CRE 감염증의 발생 현황과 관련 요인을 종합적으로 분석함으로써, 기관 규모와 병동 유형을 불문한 감염감시와 예방 전략의 필요성을 뒷받침하는 근거를 제공하였다. 이는 CRE 감염관리의 관심을 종합병원 일반병동으로 확장해야 하며, 일반병동 환자를 대상으로 한 고위험군 선별과 감염예방 전략의 수립이 필요함을 강조한다. 또한 본 연구는 병동 간호사를 대상으로 한 CRE 감염관리 교육 프로그램 개발의 근거를 제공하였다. 일반병동 CRE 고위험 환자의 조기 식별, 접촉주의 적용의 적시성, 침습적 처치 시 감염예방 등을 포함하는 간호사 역량 강화 교육은 일반병동에서의 CRE 전파를 효과적으로 차단하는 데 기여할 수 있을 것으로 여겨진다.

본 연구의 제한점은 다음과 같다. 첫째, 본 연구는 단일 기관에서 수행된 후향적 조사연구로서, 그 결과를 다른 종합병원 내과 환자군으로 일반화하는 데에는 제약이 있으며, 항생제 투여 용량이나 투여 기간과 같은 세부적인 임상 정보를 충분히 통제하지 못한 한계가 있을 수 있다. 둘째, 전자의무기록을 기반으로 자료를 수집하였으므로 기록의 한계로 인해 일부 변수는 불완전하거나 누락될 가능성이 있으며, 이는 일부 변수의 정확성에 영향을 주었을 가능성이 있다. 셋째, CRE 감염군의 사례 수가 상대적으로 적어 감염군과 비감염군 간 비교 분석에서 통계적 검정력이 제한되었을 가능성이 있으며, 이로 인해 일부 관련 요인의 추정치가 과대 또는 과소 평가되었을 가능성을 배제할 수 없다. 넷째, CRE 감염군 53명 중 8명(15.1%)에서 다른 MDRO가 공존하였으므로 확인된 관련 요인의 특이성에 대한 해석에 한계가 있을 수 있다. 그러나 MDRO 공존 비율이 비교적 낮아 전체 분석 결과에 미치는 영향은 제한적일 것으로 여겨진다. 향후 연구에서는 MDRO 공존 여부를 보정변수로 포함한 다변량 분석을 통해 이러한 제한점을 보완할 필요가 있다. 이러한 제한점에도 불구하고 본 연구는 종합병원의 일반병동 내과 환자를 대상으로 CRE 감염증 발생률과 분리균의 특성 및 감염증 발생의 관련 요인을 분석함으로써, 일반병동에서의 감염관리 전략 수립을 위한 기초자료를 마련한 의의를 가지고 있다.

## 결론

종합병원 일반병동 내과 환자의 CRE 감염증 발생 관련 요인은 카바페넴계 항생제 사용, PPI 사용, 최근 3개월 이내 중환자실 입원력, 기관지내시경 시행, 그리고 높은 CCI 점수로 확인되었다. 이러한 결과는 항생제 및 PPI의 적정 사용 관리, 침습적 시술 기구의 재처리 표준화, 그리고 높은 CCI 점수를 보이는 환자를 고위험군으로 설정한 선별검사 강화가 종합병원 일반병동에서 CRE 감염을 예방하기 위한 중요한 전략임을 시사한다. 더불어 중환자실 입원력이 있는 환자군은 CRE 감염에 취약한 고위험군으로 확인된 만큼, 입원 초기부터 능동감시와 접촉주의를 적용하는 선제적 관리 전략이 요구된다.

이상의 결과를 바탕으로 다음과 같이 제언하고자 한다. 첫째, 국내 ASP는 항생제의 적정 사용에 중점을 두고 있으나, 본 연구 결과에서 PPI 사용이 CRE 감염의 강한 관련 요인으로 확인되었으므로, 항생제 및 PPI의 적정 사용을 위한 통합적 관리체계를 고려할 필요가 있다. 둘째, 기관지내시경 및 nebulizer와 같은 호흡기 시술 기구의 재처리 과정에 대한 표준화와 질 관리 평가가 강화될 필요가 있다. 셋째, CCI 점수는 CRE 감염 위험과 관련되었으나 고위험군을 구분할 국내 기준치는 제시되지 않아 향후 연구에서는 다양한 환자군을 대상으로 적절한 CCI 기준값을 마련할 필요가 있다. 넷째, AI 기반 CRE 예측모형 변수 구성에 본 연구에서 확인된 위험요인을 반영하여 임상적 활용성을 강화할 필요가 있다.

## Figures and Tables

**Table 1.** Incidence of CRE Infection per 1,000 Admissions and 1,000 Patient-days (N = 53)

No. of CRE infections	Total eligible patients	CRE infection rate (per 1,000 admissions)	No. of patient-days	CRE infection rate (per 1,000 patient-days)
53	25,341	2.1	145,772	0.36

CRE = Carbapenem-resistant Enterobacterales.

**Table 2.** Characteristics of Patients with CRE Infection (N = 53)

Variables	Categories	n (%)
Bacterial species	*Klebsiella pneumoniae*	44 (82.9)
	*Escherichia coli*	3 (5.7)
	*Enterobacter cloacae*	3 (5.7)
	*Serratia marcescens*	3 (5.7)
CPE	Yes	30 (56.6)
	No	21 (39.6)
	Not tested (discharge)	2 (3.8)
Carbapenemase gene^†^ (n = 30)	KPC	22 (73.3)
	NDM	6 (20.0)
	Not tested (discharge)	2 (6.7)
MDRO	Yes	8 (15.1)
	No	41 (77.4)
	Unknown	4 (7.5)
MDRO type^‡^ (n = 8)	VRE	1 (12.5)
	ESBL	4 (50.0)
	MRPA	1 (12.5)
	MRAB	2 (25.0)

CRE = Carbapenem-resistant Enterobacterales; CPE = Carbapenemase-producing Enterobacterales; KPC = *Klebsiella pneumoniae* carbapenemase; NDM = New Delhi metallo-β-lactamase; MDRO = Multidrug-resistant organisms; VRE = Vancomycin-resistant *Enterococcus*; ESBL = Extended-spectrum β-lactamase; MRPA = Multidrug-resistant *Pseudomonas aeruginosa*; MRAB = Multidrug-resistant *Acinetobacter baumannii*. ^†^Percentages were calculated based on CPE-positive cases; ^‡^Percentages were calculated based on patients with MDRO.

**Table 3.** Differences in CRE Infection According to General and Clinical Characteristics (N = 265)

Variables	Categories	Infection (n = 53)	Non-infection (n = 212)	χ^2^ / U	*p*
Sex	Men	37 (69.8)	111 (52.4)	4.55	.033
	Women	16 (30.2)	101 (47.6)		
Age (years)	< 65	8 (15.1)	44 (20.8)	0.54	.463
	≥ 65	45 (84.9)	168 (79.2)		
Diagnosis	Respiratory	22 (41.5)	22 (10.4)		< .001
	Cardiovascular	2 (3.8)	22 (10.4)		
	Digestive	10 (18.9)	59 (27.8)		
	Renal	3 (5.7)	21 (9.9)		
	Diabetes mellitus	2 (3.8)	9 (4.2)		
	Cancer	11 (20.7)	56 (26.4)		
	Others^†^	3 (5.7)	23 (10.8)		
Length of stay (days)^‡^		33.00 [16.50, 43.75]	10.00 [7.00, 17.00]	9564.00	< .001
Discharge status	Death	14 (26.4)	14 (6.6)	15.58	< .001
	Survival	39 (73.6)	198 (93.4)		
Admission route^§^	Long-term care facility	5 (9.4)	7 (3.3)		.010
	Other hospital	5 (9.4)	11 (5.2)		
	General hospital	8 (15.1)	12 (5.7)		
	Community	35 (66.0)	181 (85.4)		
CCI score^‡^		3.00 [2.00, 5.00]	2.00 [0.00, 3.00]	7441.50	< .001
Pressure sore (present)^§^	Stage 1	1 (1.9)	1 (0.5)		.361
	Stage 2	0 (0.0)	1 (0.5)		
	Stage 3	1 (1.9)	0 (0.0)		
Nutrition stage	Low risk	7 (13.2)	69 (32.5)	15.71	< .001
	Moderate risk	34 (64.2)	128 (60.4)		
	High risk	12 (22.6)	15 (7.1)		
Surgery within 3 months	Yes	10 (18.9)	18 (8.5)	3.80	.051
	No	43 (81.1)	194 (91.5)		
ICU admission within 3 months	Yes	22 (41.5)	13 (6.1)	45.74	< .001
	No	31 (58.5)	199 (93.9)		

Values are presented as n (%) or median [interquartile range]. CRE = Carbapenem-resistant Enterobacterales; CCI = Charlson Comorbidity Index; ICU = Intensive care unit. ^†^Includes rehabilitation hospitals, and Korean medicine hospitals; ^‡^Mann–Whitney U test; ^§^Fisher’s exact test.

**Table 4.** Characteristics Related to Treatment and Invasive Procedure (N = 265)

Variables	Infection (N = 53)	Non-infection (n = 212)	χ^2^ / Fisher	*p*
Penicillin	31 (58.5)	71 (33.5)	11.19	< .001
3rd/4th-generation cephalosporin	18 (32.0)	70 (33.0)	0.02	1.000
Carbapenem	30 (56.6)	20 (9.4)	61.63	< .001
Fluoroquinolone	14 (26.0)	25 (11.8)	7.22	.013
Glycopeptide	20 (37.7)	11 (5.2)	43.48	< .001
Aminoglycoside^†^	3 (5.7)	7 (3.3)		.424
Tetracycline^†^	2 (3.8)	0 (0.0)		.039
Macrolide^†^	2 (3.8)	1 (0.5)		.103
TPN	22 (41.5)	23 (10.8)	28.27	< .001
Steroid^†^	9 (17.0)	17 (8.0)		.088
PPI	22 (41.5)	19 (9.0)	34.34	< .001
Chemotherapy	3 (5.7)	12 (5.7)	0.00	1.000
Nebulizer	18 (34.0)	15 (7.1)	28.11	< .001
Tracheostomy^†^	3 (5.7)	1 (0.5)		.026
Ventilator^†^	7 (13.2)	1 (0.5)		< .001
Nasogastric tube	21 (39.6)	6 (2.8)	62.72	< .001
Vascular catheter	30 (56.6)	46 (21.7)	25.26	< .001
Drainage catheter	23 (43.4)	52 (24.5)	7.44	.011
Urinary catheter	26 (49.1)	48 (22.6)	14.70	< .001
Hemodialysis	6 (11.3)	13 (6.1)	1.72	.229
Bronchoscopy	18 (34.0)	11 (5.2)	36.02	< .001
EGD	8 (15.1)	47 (22.2)	1.29	.344
ERCP	11 (20.8)	28 (13.2)	1.92	.242
Coronary angiography^†^	0 (0.0)	6 (2.8)		.603
Percutaneous biopsy	4 (7.5)	15 (7.1)	0.01	1.000

Values are presented as n (%). TPN = Total parenteral nutrition; PPI = Proton pump inhibitor; EGD = Esophagogastroduodenoscopy; ERCP = Endoscopic retrograde cholangiopancreatography. ^†^Fisher’s exact test.

**Table 5.** Factors Associated with CRE Infection (N = 265)

Variables	Categories	Crude OR (95% CI)	*p*	Adjusted OR (95% CI)	*p*
Sex (ref. Men)	Women	0.48 (0.25–0.91)	.024	0.63 (0.23–1.76)	.379
Admission route (ref. Other hospital^†^)	Long-term care facility	1.57 (0.33–7.48)	.570	1.76 (0.12–25.24)	.831
	General hospital	1.47 (0.37–5.86)	.588	2.02 (0.22–18.62)	.536
	Community	0.43 (0.14–1.30)	.134	0.56 (0.10–3.19)	.510
CCI score		1.30 (1.15–1.47)	< .001	1.36 (1.10–1.69)	.005
Nutrition stage (ref. Low)	Intermediate	2.62 (1.10–6.22)	.029	2.47 (0.60–10.10)	.208
	High	7.89 (2.66–23.37)	< .001	6.12 (0.90–41.83)	.065
ICU admission within 3 months (ref. No)	Yes	11.91 (5.38–26.38)	< .001	6.23 (1.27–30.58)	.024
Fluoroquinolone		2.69 (1.28–5.63)	.009	1.12 (0.28–4.47)	.872
Glycopeptide		11.07 (4.86–25.21)	< .001	1.21 (0.22–6.75)	.827
Penicillin		2.80 (1.51–5.18)	.001	3.64 (1.21–10.92)	.021
PPI		7.21 (3.50–14.83)	< .001	7.62 (2.27–25.56)	.001
Carbapenem		12.52 (6.14–25.52)	< .001	8.10 (2.02–32.41)	.003
TPN		5.83 (2.90–11.71)	< .001	2.31 (0.70–7.66)	.170
Nebulizer		6.75 (3.12–14.64)	< .001	1.18 (0.29–4.73)	.818
Bronchoscopy		9.40 (4.09–21.59)	< .001	5.31 (1.07–26.42)	.042
Nasogastric tube		22.42 (8.41–59.79)	< .001	4.80 (0.95–24.31)	.058
Urinary catheter		3.38 (1.80–6.34)	< .001	1.90 (0.60–6.00)	.275
Vascular catheter		4.71 (2.50–8.87)	< .001	0.50 (0.13–1.97)	.324
Nagelkerke R^2^ = .67, Cox & Snell R^2^ = .43					

The references for all binary treatment and invasive procedure variables were set as “No.” OR = Odds ratio; CI = Confidence interval; ref. = Reference; CCI = Charlson Comorbidity Index; ICU = Intensive care unit; PPI = Proton pump inhibitor; TPN = Total parenteral nutrition. ^†^Includes long-term care hospitals, rehabilitation hospitals, and Korean medicine hospitals.
